# Development and Characterization of a Sol–Gel-Functionalized Glass Carbon Electrode Probe for Sensing Ultra-Trace Amounts of NH_3_ and NH_4_^+^ in Water

**DOI:** 10.3390/gels10060382

**Published:** 2024-06-04

**Authors:** H. Alwael, M. Oubaha, M. S. El-Shahawi

**Affiliations:** 1 Department of Chemistry, Faculty of Science, King Abdulaziz University, P.O. Box 80203, Jeddah 21589, Saudi Arabia; 2 Centre for Research in Engineering Surface Technologies (CREST), FOCAS Institute, Technological University Dublin, 13 Camden Row, D08 CKP1 Dublin, Ireland; am.oubaha@gmail.com

**Keywords:** Berthelot’s reaction, detection of NH_3_ and/or NH_4_, modified glassy carbon electrode, sol–gel, stripping voltammetry, water samples

## Abstract

This study centers on the development and characterization of an innovative electrochemical sensing probe composed of a sensing mesoporous functional sol–gel coating integrated onto a glassy carbon electrode (sol–gel/GCE) for the detection of NH_3_ and/or NH_4_^+^ in water. The main interest for integrating a functional sol–gel coating onto a GCE is to increase the selective and sensing properties of the GCE probe towards NH_3_ and/or NH_4_^+^ ions. The structure and surface morphology of the newly developed sol–gel/GCE probe were characterized employing scanning electron microscopy (SEM), atomic force microscopy (AFM), dynamic light scattering (DLS), and Fourier-transform infrared (FTIR), while the electrochemical sensing properties were evaluated by Berthelot’s reaction, cyclic voltammetry (CV), and adsorptive square wave–anodic striping voltammetry (Ads SW–ASV). It is shown that the newly developed sol–gel coating is homogeneously deposited on the GCE with a sub-micron and uniform thickness close to 630 nm and a surface roughness of 25 nm. The sensing testing of the sol–gel/GCE probe showed limits of detection and limits of quantitation of 1.7 and 5.56 nM of NH_4_^+^, respectively, as well as a probe sensitivity of 5.74 × 10^−1^ μA/μM cm^−2^. The developed probe was fruitfully validated for the selective detection of NH_3_/NH_4_^+^ in fresh and sea water samples. Computed Student *t_exp_* (0.45–1.25) and *F_exp_* (1.69–1.78) (*n* = 5) tests were less than the theoretical *t_tab_* (2.78) and *F_tab_* (6.39) at 95% probability.

## 1. Introduction

The current global water crisis emanating from the discharge of industrial effluents into natural water without treatment has attracted comprehensive attention and criticism [1]. Water pollution has dramatically increased due to the growth of diverse industries such as photoengraving, leather tanning, wood treatment, paper pulp, glass, and ceramic production [1,2], which have led to the increased uptake of nitrogen-containing compounds in surface and groundwater [1]. Dissolved ammonia is an environmentally significant component of the nitrogen cycle. Moreover, aqueous ammonia not only causes an increase in the eutrophication of water but is also a crucial threat to drinking water quality and larval aquatic products [2,3,4]. Indeed, in the ground and surface water, ammonia can be present at 10 µg/L and up to 30 µg/L in wastewaters [5].

Numerous techniques, including ion chromatography [4,5,6,7], HPLC [8,9], and gas chromatography [10,11], have been reported for the determination of NH_4_^+^ in water. In chromatographic techniques, the main problems are the HPLC optical detector and mass spectrometers coupled with GC that are not sensitive enough to detect NH_3_/NH_4_^+^ in water [11,12]. Hence, derivatization steps are frequently employed to change NH_4_^+^ into a suitable form for precise HPLC or GC detection [13]. NH_4_^+^ is normally derivatized with *o*-phthalaldehyde (OPA) in HPLC to give an intensely fluorescent azaindole, whereas in GC mass spectrometry methods, an alkyl chloroformate such as butyl chloroformate is used to convert NH_4_^+^ ions to higher molecular mass derivatives [10,11,12]. Other optical detection methods such as spectrophotometry [5,14,15,16], fluorescence-based emission [17,18] and colorimetry [19] have been reported for the detection of NH_4_^+^ in environmental water samples. However, most of these techniques suffer from many drawbacks such as expensive equipment and elaborated sample pre-treatment; hence, it is of great importance to develop an efficient and cost-effective method for the detection of NH_3_/NH_4_^+^ [17,18].

Nowadays, designing and constructing electrochemical sensors for the precise detection and monitoring of NH_3_ and/or NH_4_^+^ have received great interest [20]. Surface-modified electrodes (SMEs) have been investigated as potential alternatives to Hg electrodes and bare glassy carbon electrodes [21,22], with promising results being achieved. SME-coupled stripping methods are a successful approach in addressing the shortcomings in current methodologies of trace analysis of various complex species [20,23,24,25]. However, most of these methods suffer from critical limitations, including low sensitivity and matrix interference. To sort out these limitations, a great devotion has been oriented towards applications of the SME with nanosized metals, metal oxides, sol–gels, and other bare electrodes [25,26]. Indeed, numerous studies involving noble metals including Ag, Au, Ni, and Pt, and metal oxides such as CuO, ZnO, and *α*-Fe_2_O [27,28,29] nanoparticles have been developed for the detection of NH_3_ and/or NH_4_^+^ at trace levels in water. However, most of these materials showed high consumption, low stability, and low selectivity for the following reasons: (i) an irreversible agglomeration of Ag nanoparticles in high pH solution, high consumption and short service life of Ag nanoparticles, and (ii) the sensing materials must be sprayed or pressed onto conductive substrates via binders compared to binder-free electrodes. Therefore, the development of novel materials including sol–gel coatings with high specific surface areas (SSAs), greater stability, and ease of process has been highly recommended [30,31,32].

Indeed, the sol–gel process provides a convenient and economical route to create advanced organic–inorganic hybrid materials with tunable selectivities and sensitivities, controllable morphologies, and high thermal and chemical stabilities [33,34]. Sol–gel-derived polymer-modified silica have been used for environmental remediation due to their availability, high SSAs, easy surface functionalization, and ability to capture specific pollutants [35,36,37]. To the best of our knowledge, no study has to date involved electrochemical determination of NH_3_ and/or NH_4_^+^ on sol–gel/GCE or other surface-modified electrodes, particularly those based on Berthelot’s reaction [22]. Additionally, Ads SW–ASV accompanying the accumulation steps to enrich the analyte into or onto the modified electrode that is oxidized in the current measurement step can indorse the analytical sensitivity [22,36]. With this background in mind, a sol–gel-functionalized GCE probe may be an ideal and sustainable approach for the detection of NH_3_ and/or NH_4_^+^. Therefore, the current study centers on (i) studying the redox characteristics of the electroactive species of Berthelot’s reaction (Electronic Supplementary Information, ESI. 1) using sol–gel-modified GCE for developing an expedient, rapid, and robust Ads SW–ASV-viable and straightforward electrochemical approach for the detection of trace levels of NH_3_ and/or NH_4_^+^ based on Berthelot’s reaction combining sol–gel/GCE; (ii) assigning the most probable electrochemical oxidation mechanism of Berthelot’s reaction (indophenol); and (iii) testing the impact of interfering inorganic and other organic species likely present in water samples.

## 2. Results and Discussion

### 2.1. Characterization of the Sol–Gel/GCE

#### 2.1.1. Structure and Surface Morphology of the Fabricated Sol–Gel/GCE

A highly chemically resistant sol–gel coating based on a suitable combination of hybrid silicon and zirconium precursors was prepared to withstand chemically aggressive environments such as those imposed by ammonia and ammonium, as detailed in Section 4.3. The characterizations of the structure and surface morphology of the prepared sol–gel material and sol–gel/GCE were performed to properly identify the effective occurrence of the sol–gel reactions and suitable deposition of the coating onto the substrate.

**Figure 1:**
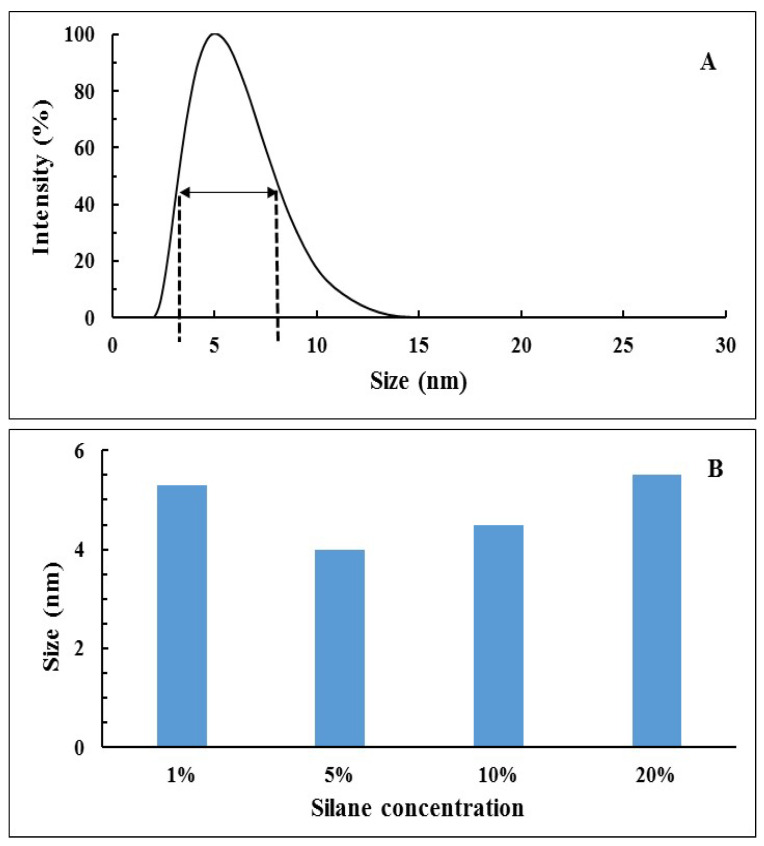
DLS measurements for APTES sol–gel (**A**) and full width at half maximum (FWHM) (**B**) for different silane concentrations.

The particle size of the synthesized sol–gel material was critically recorded within 60 min after preparation using dynamic light scattering (DLS, Figure 1A). The DLS spectrum exhibits one well-defined distribution band in the range of 0–20 nm and centered at 5.0 nm (±1 nm). To better understand the distribution of the particle size within the material, the full width at half maximum (FWHM) was measured by recording the DLS spectra at various silane concentrations (1.0–20.0%) and the obtained data are illustrated in Figure 1B. The FWHM was found to be equal to 5.1 nm, revealing that the majority of the particle sizes are close to the maximum recorded particle size of 5 nm. For other concentrations of functional silanes (5, 10, and 20%), the maximum particle size varies around 5.0 nm (Figure 1B). Thus, the addition of APTES at up to a concentration of 20% does not significantly alter the particle size of the material.

**Figure 2:**
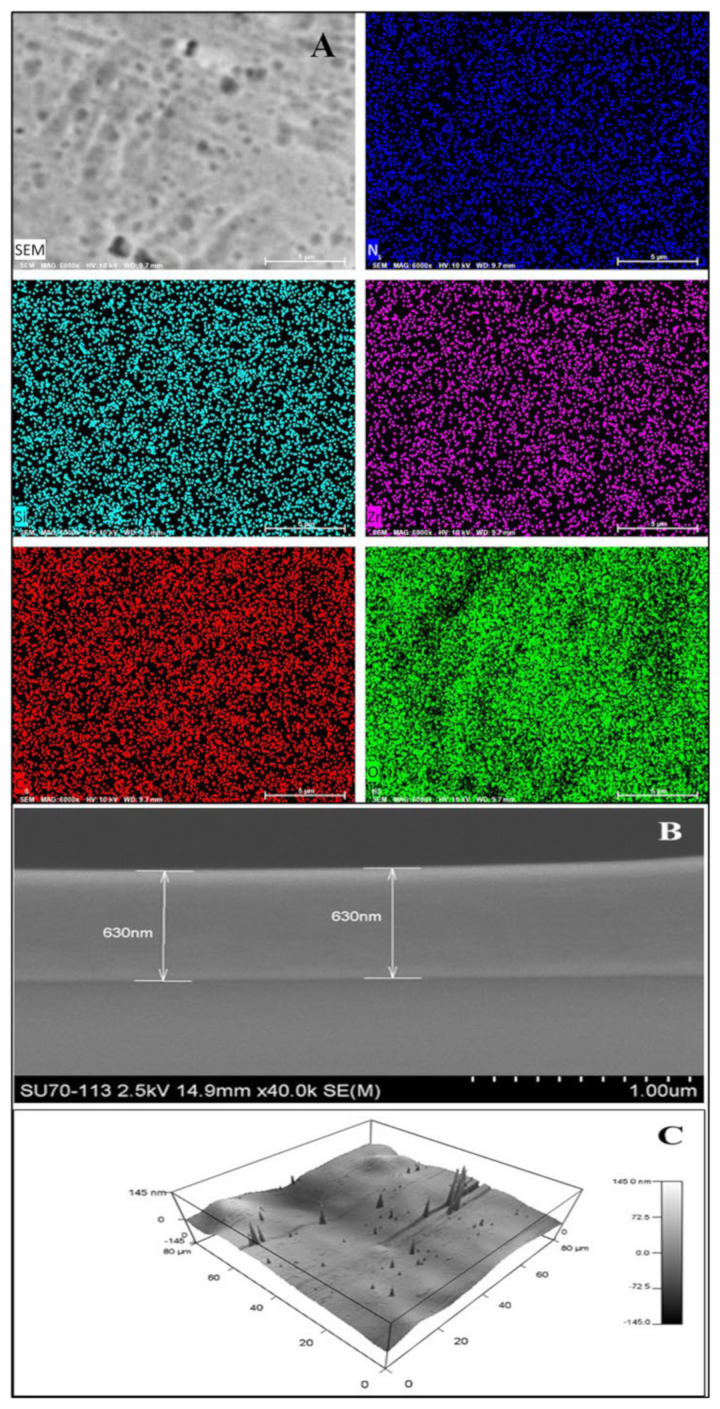
Top view SEM image and EDX analysis of the sol–gel coating on the bare GCE (**A**); cross-section view of the sol–gel-modified GCE (scale bar = 5 µm); (**B**); and top view AFM image of the sol–gel-modified GCE (**C**).

The SEM images of the APTES based sol–gel coating on the bare GCE were recorded to characterize the structure and surface morphology (Figure 2A) and cross-section (Figure 2B). In addition, EDX analysis was performed to identify the presence and homogeneity of the various chemical elements in the coating. Figure 2A shows that the surface of the coating exhibits a typical honeycomb morphology with submicron pores, typically ranging from a few tens to several hundreds of nm. EDX analysis of the surface of the coating shows a uniform distribution of the main elements contained in the employed sol–gel precursors, namely oxygen, carbon, nitrogen, silicon, and zirconium. The uniform distribution of these chemical species within the coating is a clear indication of the good homogeneity of the coating in terms of chemical composition. In addition, the cross-section view of the coating (Figure 2B) shows that the sol–gel coating is uniformly deposited on the bare GCE with a continuous thickness of 630 nm ± 10 nm. Achieving uniform coatings with controllable thicknesses is essential for sensing applications, as those will define the content of active sensing sites and reproducibility of the sensing performances of the platform. This demonstrates the successful deposition and adhesion of the sol–gel coating onto the GCE surface with no delamination or cracks, translating a suitable material affinity between the substrate and the coating. It is worth noting that despite the very high dilution of the sol–gel material (99%), a coating layer can still be detected, suggesting a high reactivity of the sol–gel coating with the surface of the GCE. Therefore, the SEM and EDX results concord to demonstrate that the morphology of the sol–gel coating is clearly porous, thus definitely increasing the surface area on top of the GCE. Nevertheless, in order to further characterize the morphology of the coating, AFM analysis was performed and presented in Figure 2C. Here, one can observe that the surface exhibits an average roughness of 25 nm ± 5 nm and some peaks with heights of up to a few tens of nm. It can be safely proposed that the peaks observed at the surface are due to the solvent evaporation phase, which carries away parts of the sol–gel material that subsequently solidifies on top of the coating surface.

The FTIR spectrum of the sol–gel coating shown in ESI. 2 displayed well-defined vibration bands at 1646 (N-H), 1562 (N-H), 1400 (Si-C), 1350 (Si-C), 1150 (Si-O-Si), 1050 (Si-O-Si), 875 (Si-OH), 795 (Si-OH), and 711 cm^−1^ (C-H). The absence of the ethoxy-silane band (Si-O-C_2_H_5_) at 1170 cm^−1^ and the presence of the Si-OH band demonstrates that APTES has undergone full hydrolysis. The presence of the two silicate vibrations at 1050 and 1150 cm^−1^, along with the presence of the silanol vibration, proves that sol–gel condensation reactions have taken place, thus leading to the formation of a silicate backbone containing reactive silanol groups. The presence of N-H vibrations suggests that the hydrolysis process does not alter the amine functionalities and that they are located with the silicate network. Therefore, the FTIR results concord to demonstrate that the hydrolysis and condensation reactions have successfully led to the preparation of a hybrid silicate coating containing both amine and silanol functionalities available for potential reactions with biomolecules, indophenol, NH_3_, and NH_4_^+^. Finally, the low resolutions of some vibrations are characteristic of submicron coating (as shown by SEM analysis above), as demonstrated elsewhere [34,35,36].

#### 2.1.2. Electrochemical Characterization

To explore the active surface area of the GCE and the sol–gel/GCE as working electrodes, the CVs of the reversible electrochemical probe K_3_[Fe(CN)_6_] (1.0 × 10^−3^ M) in the potential range 0.0–1.0 V at different sweep rates (ν) in KCl (0.1 M) as electrolyte versus Ag/AgCl electrode were recorded at various sweep rates (v). The surface area (A, cm^2^) of the working electrode was computed using the Randles–Sevcik equation [37,38]:

i_p,a_ = (2.69 × 10^5^) n^3/2^AC_o_D^1/2^v^1/**2**^ (1)

where i_p,a_ refers to the anodic peak current, n is the number of electron(s) transferred, D and C_o_ are the diffusion coefficient (cm^2^ s^−1^) and concentration (1.0 × 10^−3^ M) of the redox probe K_3_[Fe(CN)_6_], and ν is the sweep rate (Vs^−1^). For K_3_[Fe(CN)_6_] (1.0 × 10^−3^ M) containing KCl (1.0 × 10^−1^ M) as electrolyte, n = 1 and D = 7.6 × 10^−6^ cm^2^ s^−1^ [35,36,37]. The results are illustrated in ESI. 3. The microscopic active surface areas of the bare GCE and sol–gel/GCE as computed from the slopes of the linear plots of i_p,a_ versus the square root of the sweep rate (ν^1/2^) were found to equal 0.0077 and 0.0168 cm^2^, respectively. The relative microscopic active surface area of the sol–gel/GCE increased by 2.18 times the corresponding value of the bare GCE. This accounts for the advanced conductivity of the developed sensing probe and improved the electron transfer process between the electrode couple [Fe(CN)_6_]^4−^/[Fe(CN)_6_]^3−^ and the working electrode surface.

### 2.2. Redox Behavior

**Scheme 1:**
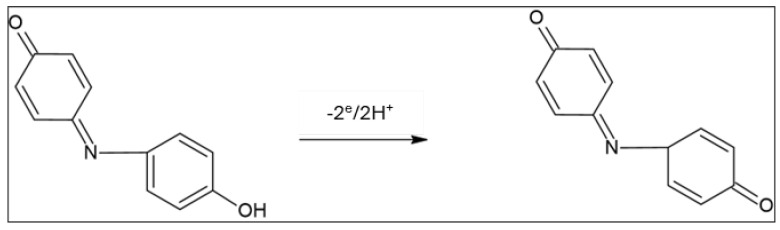
A proposed electrochemical oxidation mechanism for the developed reaction product (indophenol) of Berthelot’s reaction at the sol–gel/GCE vs. Ag/AgCl electrode.

**Figure 3:**
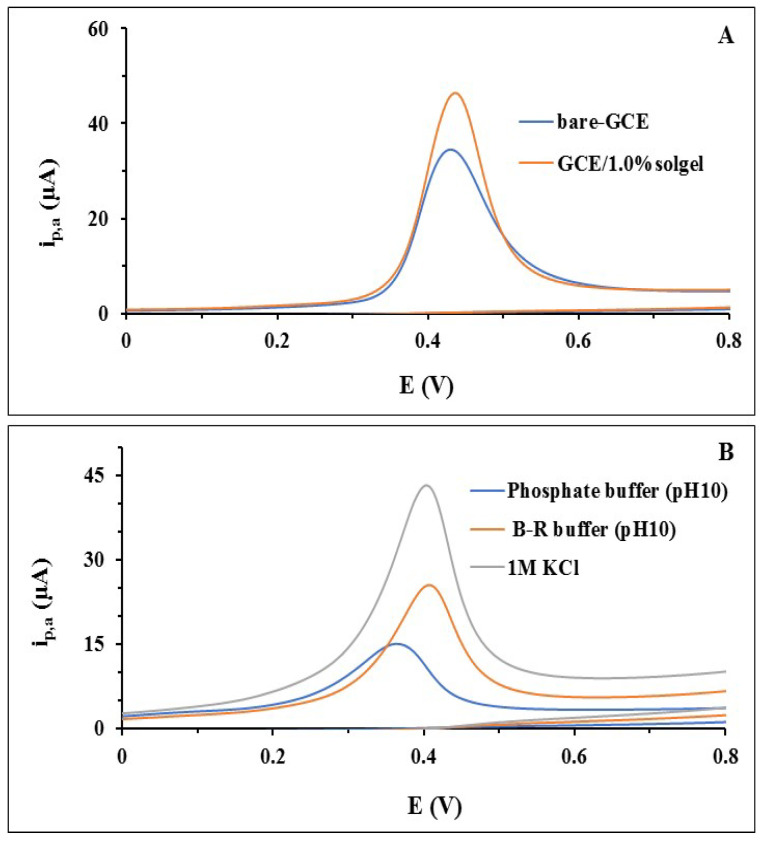
CVs of NH_4_^+^ (5.55 × 10^−4^ M) in KCl (1.0 M) at bare GCE and sol–gel/GCE (**A**) and in the presence of various supporting electrolytes at sol–gel/GCE (**B**) at 50 mVs^−1^ versus Ag/AgCl electrode.

The CV responses of the sol–gel/GCE and bare GCE in the presence of NH_3_/NH_4_^+^ (5.55 × 10^−4^ M) at KCl (1.0 × 10^−1^ M) were recorded. The CVs at 50 mV s^−1^ sweep rate versus the Ag/AgCl electrode are demonstrated in Figure 3A. In the potential window from 0.0 to 1.5 V, the CV of the background supporting electrolyte at bare GCE displayed no peaks in the forward and reverse scan at the tested scan rates (50–100 mVs^−1^), whereas in the presence of NH_3_/NH_4_^+^, one well-defined anodic peak at E_p,a_ = 0.40 V was observed, as illustrated in Figure 3A. Similarly, the sol–gel/GCE probe, in the presence of NH_3_/NH_4_^+^ under the same experimental conditions, also revealed one well-defined oxidation peak but located at a higher peak potential (0.48 V). Additionally, the CV response of NH_3_/NH_4_^+^ was also found to increase from 35 to 48.0 μA. This remarkable enhancement on the electrochemical current response of NH_3_/NH_4_^+^ achieved by the sol–gel/GCE is most likely due to the increase in the surface area and functionality of the sol–gel coating. Indeed, as identified by the FTIR analysis and EDX results above, the sol–gel coating contains both hydrophilic groups (silanol and amine groups) and electrophilic groups (zirconium species) that can favorably interact with both electrophilic and nucleophilic groups, respectively. However, thanks to the nucleophilic character of the reactive oxidized indophenol product (see Scheme 1 below), the preferential sensing mechanism involves a nucleophilic attachment via the filling of the d free orbitals of the zirconium atom by the free pairs of electrons available on the two oxygen atoms and the free pair of electrons on the nitrogen atom of the indophenol reactive product, ultimately leading to the formation of ligands.

As the nature of the supporting electrolyte may intensely impact the stability of the electrochemical species of the target species, the CVs were recorded in different electrolyte buffers, including B-R buffer at pH 10, phosphate buffer at pH 10, and KCl at pH 10 (Figure 3B). It can be seen that the best voltametric signal was obtained by employing KCl as a supporting electrolyte. Additionally, the affinity of the oxidation product of indophenol and its stability towards the sol–gel/GCE are likely accounting for the observed trend in the KCl electrolyte. Thus, KCl was selected as a reference electrolyte at pH 10 in this study.

**Figure 4:**
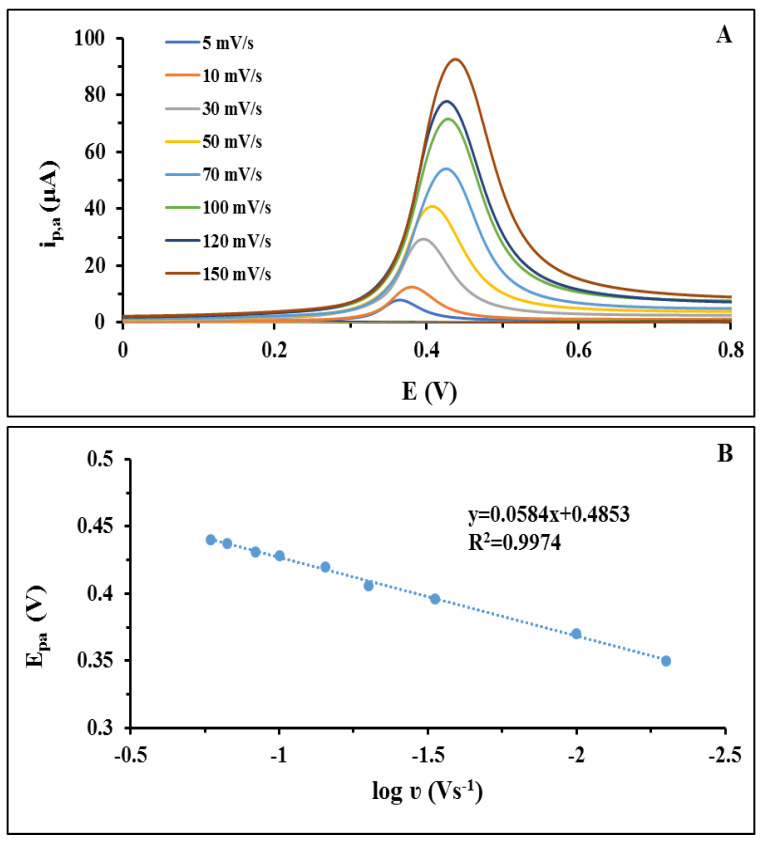
CV of the produced indophenol at a [NH_4_]^+^ concentration of 5.55 × 10^−4^ M in KCl at various sweep rates (5–150 mVs^−1^) (**A**) and plot of E_p,a_ versus log ν (**B**) of indophenol resulting from NH_4_^+^ (5.55 × 10^−4^ M) in KCl (1.0 M) electrolyte.

To explore the redox behavior of the indophenol, the CVs of Berthelot’s reaction product (indophenol formation) were recorded in the KCl electrolyte at pH 10 and at various sweep rates (ν = 5.0–150 mVs^−1^) versus the Ag/AgCl electrode. The obtained CVs of the indophenol on the sol–gel/GCE are presented in Figure 4A. The CV displayed a well-defined oxidation peak at all sweep rates in the range 0.35–0.44 V, whereas when the scan is reversed, no cathodic peak was observed, confirming the irreversible nature of the electrochemical process. Moreover, by increasing the scan rate from 5 to 150 mVs^−1^, the potential of the anodic peak was shifted to higher potential values, further supporting the irreversible nature of the electrochemical process. The plot of log anodic peak current (log i_p,a_) corresponding to the indophenol oxidation product of Berthelot’s reaction vs. log sweep rate (ν) was linear (R^2^ = 0.9995), with a slope of 0.7259 (ESI. 4). The linear plot can be expressed by the regression equation:

log i_p_ (μA) = 0.7259 + 0.386 log ν (mVs^−1^) + 0.9981; R^2^ = 0.9934 (2)

The slope (0.7259) of the linear plot was found higher than > 0.5, adding extra support to the irreversible nature of the oxidation peak of indophenol. Further, on growing the sweep rate, the potential of the anodic peak shifted to more positive potential values and the plot of the peak potential versus log ν was linear (Figure 4B). The plot can be stated by the following equation:

E_p,a_ (V) = 0.0584 log ν (V s^−1^) + 0.4853; R^2^ = 0.9974 (3)

**Figure 5:**
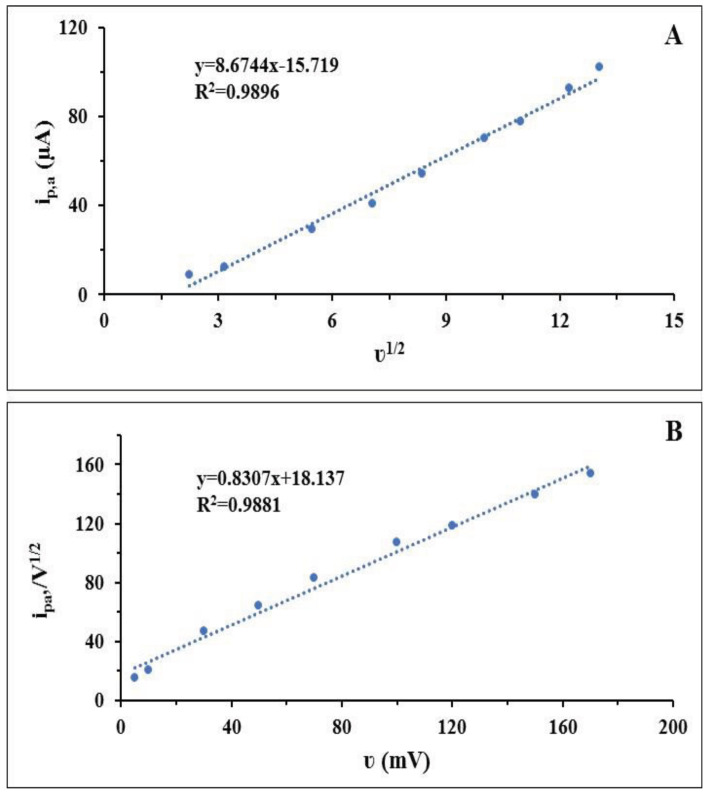
Plots of i_p,a_ versus ν^1/2^ (**A**) and the current function (i_p,a_/ν^1/2^) versus ν (**B**) of indophenol (5.55 × 10^−4^ M) in KCl (1.0 M) as supporting electrolyte at pH 10.

According to Laviron’s model [39], the anodic peak potential (E_p,a_) of the irreversible electrode process can be defined by the following equation:

(4) $$E_{p}=E{}^{\circ}+\frac{2.303RT}{\alpha nF}log\frac{RTK{}^{\circ}}{\alpha nF}+\frac{2.303RT}{\alpha nF}\log\nu$$

where α is the electron transfer coefficient, K^°^ is the standard heterogeneous rate constant of the electrode reaction, and the other signs have their normal meanings. The αn value was simply computed from the slope of the linear plot of E_p,a_ vs. log ν. Taking T = 298 K, R = 8.314 J K^−1^ mol^−1^, F = 96,480 C, and the value of the slope as 0.0584 (Figure 4B), the calculated value of αn was found equal to 1.01, anticipating an irreversible electrode process with 2H^+^/2e transfer. The value of the standard heterogeneous rate constant (K^°^) was also computed from the intercept of the linear plot of the peak potential versus log ν (Figure 4B). The E^°^ value can be obtained from the intercept of the linear plots of E_p_ vs. ν by extrapolating to the vertical axis at ν = 0 [40,41]. The intercept and E^0^ of the developed anodic peak as computed from Figure 4B were found to be 0.4853 and 0.31, respectively. The corresponding K^°^ value was also evaluated as K^°^ = 3.9 × 10^4^ s^−1^, supporting the oxidation of indophenol via the 2H^+^/2e electrochemical step at the sol–gel-modified GCE. Thus, the oxidation process can be expressed by Scheme 1, as follows:

Exploring the redox behavior of the electrochemical oxidation of indophenol was further assigned by studying the impact of the square root of the sweep rate (ν^1/2^) on the anodic peak current. By increasing the sweep rate, the anodic peak current increased linearly and the plot of the i_p,a_ versus ν^1/2^ was found to be linear (Figure 5A). The plot of i_p,a,_ versus ν^1/2^ at the established sol–gel/GCE probe can be expressed by the following regression equation:

i_p,a_ (V) = 8.6744 ν^1/2^ (mV s^−1^)^1/2^ − 15.719; (R^2^ = 0.9896) (5)

Thus, the electrochemical oxidation of the indophenol process is a diffusion-controlled irreversible oxidation process, as reported earlier [40,41].

The impact of the sweep rate on the anodic peak current function (i_p,a_/ν^1/2^) of the electrochemical oxidation of indophenol at the sol–gel-modified GCE was further studied (Figure 5B). By increasing the sweep rate, the current function (i_p,a_/ν^1/2^) increased continuously, signifying that the oxidation of the OH group of the indophenol does not favor the EC-type mechanism [38,40]. Indeed, in an EC mechanism with an irreversible electrode process, the ratio of the current function (i_p,c_/ν ^½^) decreases constantly when increasing the scan rate [38,40].

The plot of the anodic peak current vs. sweep rate at sol–gel-modified GCE was found to be linear (ESI. 5), and it can be expressed by the following regression equation:

i_p,a_ (μA) = 0.5584 v (mV s^−1^) + 10.467; R^2^ = 0.9902 (6)

The surface coverage (Γ) of the electroactive species of indophenol was further calculated employing the following equation [38,40]:

i_p,a_ = n^2^F^2^A Γ ν/4RT (7)

where n = number of electron transfer participating in the electrode process, and the other symbols have their regular meanings. The plot of i_p,a_ vs. the scan was linear, as displayed in ESI. 5. Assuming n = 2, the computed value of Γ of indophenol was found equal to 8.85 × 10^−6^ mol cm^−2^. The high value of Γ (8.85 × 10^−6^ mol cm^−2^) recommended the possible use of the developed sensing probe (sol–gel/GCE) for trace and ultra-trace determinations of NH_3_ and/or NH_4_^+^ ions in water using Ads SW–ASV.

### 2.3. Optimization of the Analytical Parameters of the Ads SW–ASV Assay

The Ads SW–ASV offers many advantages over the common electrochemical techniques, e.g., CV, normal pulse voltammetry (NPV), and DPV, since the waveform is a series of pulses growing along a linear baseline, whereas the current signal is measured in a forward pulse and reverse pulses [22,38]. As the efficiency of the established sol–gel/GCE may be affected by both the coating fabrication conditions (sol–gel concentration and deposition time) and the electrochemical sensing parameters (nature of the supporting electrode, accumulation time, deposition potential, pulse amplitude, frequency, and sweep rate), these critical parameters are individually studied in detail below.

**Figure 6:**
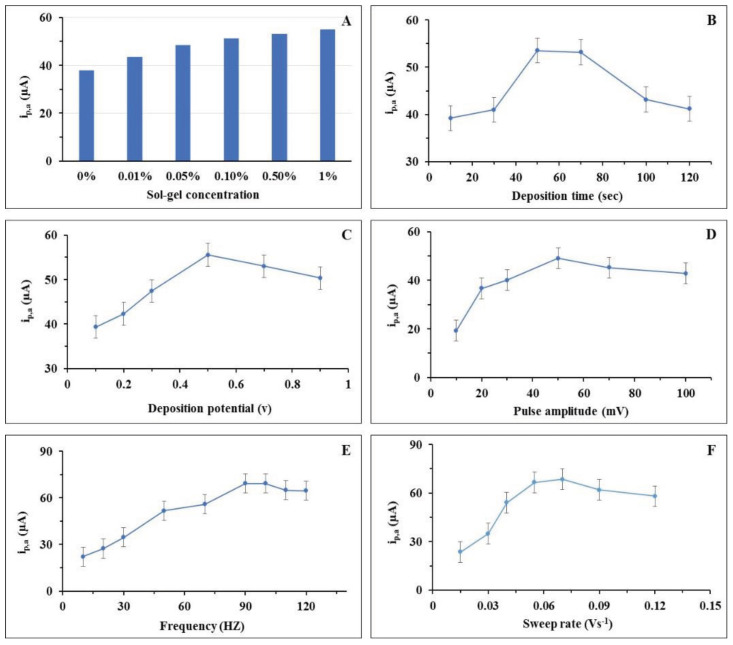
Impact of the sol–gel concentrations (**A**), deposition time (**B**), accumulation potential (**C**), pulse amplitude (**D**), frequency (**E**), and sweep rate (**F**) on the i_p,a_ (at 0.42 V) of the oxidation product of indophenol versus Ag/AgCl electrode. Conditions: [NH_4_^+^] = 2.8 × 10^−6^ M; solution pH = 10; supporting electrolyte = KCl (1.0 M).

*Influence of the supporting electrolyte*: The Ads SW–ASV of indophenol at pH 10 were recorded in numerous buffer solutions including B-R buffer (pH 10), phosphate buffer (pH 10), and KCl (1.0 M) at pH 10 adjusted with a few drops of NaOH (0.05 M). Well-defined peaks and maximum anodic peak currents were obtained in the KCl (1.0 M) electrolyte solution (ESI. 6), in agreement with the CVs mentioned above at pH 10 (Figure 3B). These results suggest that KCl is most likely enhancing the electrolysis of the oxidation product of indophenol and facilitating the H^+^/e^−^ transfer, probably by generating a higher polarity than the other electrolytes. Thus, KCl is adopted as the optimal electrolyte in the subsequent study.
*Influence of the sol–gel concentration*: The sol–gel concentration (0.01–1.0% *v*/*v*) on the Ads SW–ASV response was critically studied (Figure 6A), as this is likely affecting the thickness of the coating, and thus its surface area. One can observe that the untreated GCE exhibits an anodic peak current of 37 μA and the integration of the sol–gel coating at a concentration of 0.01% enables a direct increase to 42 μA. The further increase of the sol–gel concentration provokes a regular increase of the anodic peak current that reaches the highest value of 55 μA for the coating prepared with a sol–gel concentration of 1%. This result translates the direct impact of the sol–gel coating on the probe sensitivity, which is believed to be due to the increased interaction between the analyte and the probe, certainly enabled by the increase in the surface area and higher chemical interaction with the sol–gel. Owing to these results, it has been decided to pursue the rest of the sensing testing by employing the most sensitive sol–gel coating, namely the one prepared with a concentration of 1%.
*Influence of the dipping time of the GCE within the sol–gel*: The impact of the dipping time (1–5 min) of the GCE during the deposition of the sol–gel coating is proposed in ESI. 7. It can be seen that the anodic current remains stable at up to 2 min of dipping (50 μA), followed by a 10% increase after 3 min of dipping to reach a value of 55 μA. From the 3 to 5 min dipping times, the anodic current remains quite stable within the error measurement. As the concentration of the sol–gel is constant at 1%, the impact on the coating thickness is negligible. However, it is well known that the morphology of the sol–gel coatings evolves within time, with porosities evolving from macroporous to mesoporous structures with ageing time. This result signifies that the maximum anodic peak current is favored with denser coatings, where the analyte may be entrapped in a closer area to the probe. It is also worth mentioning that after a 3.0 min dipping time, a 2.0 min drying time under an IR lamp was found necessarily to achieve a stable and homogeneous sol–gel film-modified GCE. Therefore, in the subsequent study, a 3.0 min dipping time will be maintained.
*Influence of the accumulation time*: The effect of the accumulation time on the anodic peak current has been investigated in the range 10–120 s, with a fixed E_p,a_ at 0.42 V (Figure 6B). One can see that the deposition time provokes an increase in the i_p,a_ from typically 40 μA (±3 μA) at 10 and 30 s up to the maximum value of 54 μA (±3 μA) recorded at 50 s. The anodic current is stable at 70 s, then undergoes a decrease at 100 and 120 s with values similar to those recorded at 10 and 30 s. The observed behavior suggests that the implied electrochemical process is clearly time-dependent and can be explained either by an effect of the morphology of the sol–gel coating on the ability of the electrolyzed species to diffuse towards the GCE interface or on the chemistry of the sol–gel coating that may provoke time-dependent chemical reactions or a combination of both morphological and chemical effects. Indeed, as the sol–gel coating is inherently acting as a mesoporous barrier, the diffusion of the electrolyzed species would require a favorable path within the coating that will enable its efficient diffusion towards the GCE interface. On the other hand, the sol–gel coating contains a number of chemical functionalities (amine, zirconium, and silicon species) that have the ability to react with the indophenol-oxidized product. For example, the zirconium atom contains d free orbitals that are capable of hosting free pairs of electrons, such as those contained in the indophenol-oxidized products, thus forming a sol–gel surface functionalized with indophenol compounds. Therefore, this process may be time-dependent, and once completed, the diffusion of the electrolyzed compounds may take place properly. These hypotheses can explain the increase in the anodic current up to 50 s, but the decrease in the anodic current can only be explained by a decrease in the diffusion ability of the electrolyzed species, probably due to steric hindrance effects due to the filling of the mesoporous sol–gel coating and its inability to further host electrolyzed species. Nevertheless, these results have added further confirmation that the sol–gel coating has a high surface area and a likely reactivity with the electrolyzed species. Owing to these findings, the deposition time of choice is 50 s and will be adopted in the next study.
*Influence of the deposition potential*: The effect of the deposition potential on the stripping anodic current response (i_p,a_) is investigated in the range 0.1–0.9 V, as shown in Figure 6C. One can see that the anodic current increases progressively from 39 μA, recorded at 0.1 V, to a maximum value of 55 μA, recorded at 0.5 V. The anodic current is then seen to stabilize within the error bar until 0.9 V. It is believed that the deposition potential impacts the polarizability of the electrolyzed species, and thus their ability to efficiently diffuse within the sol–gel coating. This assumption is coherent with the fact that the anodic current remains stable (within the error bar) once the optimum potential of 0.5 V is attained. Therefore, the optimum deposition potential of 0.5 V will be adopted in the subsequent work.
*Influence of the pulse amplitude*: The influence of the pulse amplitude on the Ads SW–ASV peak of indophenol was examined within the range 20–100 mV (Figure 6D) under the optimal conditions defined above. It can be seen that the anodic current increases progressively from 20 μA, recorded at 20 mV, up to the maximum value (49 μA), displayed at a pulse amplitude of 50 mV. The anodic current is seen to stabilize within the error bar at up to a pulse amplitude of 100 mV. The optimum pulse amplitude of 50 mV will be used in the next work.
*Influence of the frequency*: The impact of the frequency on the anodic peak current was investigated in the range 10–120 Hz and at a peak potential of 0.42 V (Figure 6E). One can see that the anodic current increases progressively from 20 μA, recorded at 10 Hz, to the maximum value of 70 μA, recorded at 90 Hz. The anodic current is then stable within the error bar until the maximum used frequency of 120 Hz. In addition, at the optimum frequency of 90 Hz, no distortion of the peak or the baseline were noticed; thus, the frequency of 90 Hz was selected as the frequency of choice.
*Influence of the sweep rate*: The influence of sweep rate (0.015–0.120 Vs^−1^) on the i_p,a_ of ammonium ions (2.8 × 10^−6^ M) was studied under the optimal operational conditions defined above. The anodic current increases steadily upon increasing the sweep rate from 0.015–0.07 Vs^−1^ and decreases at a sweep rate higher than 0.07 Vs^−1^, as illustrated in Figure 6F. Thus, in the subsequent study, a sweep rate of 0.07 Vs^−1^ was selected, since at this sweep rate a well-defined, symmetric, and sharp anodic peak was obtained.

### 2.4. Analytical Performance

**Figure 7:**
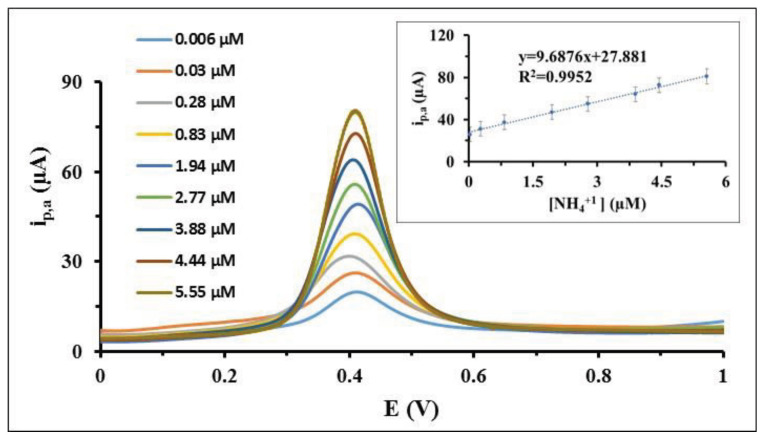
Ads SW–ASVs of indophenol resulting from various known concentrations of NH_4_^+^ (5.56 × 10^−9^–5.56 × 10^−6^ M) versus Ag/AgCl electrode at pH 10 in the KCl (1.0 M) supporting electrolyte at the optimal parameters. Inset displays calibration plot for respective concentrations of [NH_4_^+^].

Under the optimal experimental and operational parameters, the Ads SW–ASV were recorded at various known concentrations (5.56 nM to 6.0 μM) of NH_4_^+^. The results are illustrated in Figure 7. The Ads SW–ASV anodic peak current was directly proportional to the NH_4_^+^ concentrations over a wide range of concentrations (5.56 × 10^−9^–5.56 × 10^−6^ M). The plot of the anodic peak current vs. [NH_4_^+^] was found to be linear over a wide linear dynamic range (LDR) of 5.56 nM (1.0 × 10^−1^ µg L^−1^)–5.56 μM (1.0 × 10^−1^ µg mL^−1^) of NH_4_^+^ concentrations (Figure 7). The linear plot was leveled off at NH_4_^+^ concentrations higher than 5.56 μM because of the adsorption saturation of the electrochemically active species at the surface of the working electrode. The linear plot can be expressed by the following equation:

i_p,a_ (µ A) = 9.6876 C (µM) + 27.881 R^2^ = 0.9952 (8)

where i_p,a_ is the stripping voltametric peak current in µA, C is the NH_4_^+^ concentration (µM), and R is the correlation coefficient.

The calculated values of the LOD (3*σ*/*b*) and LOQ (10*σ*/*b*) [41], where *σ* is the standard deviation of five replicate measurements of the blank under the optimal parameters, and *b* is the slope (the sensitivity factor) of the linear calibration plot of the NH_4_^+^ ions, were found to be 1.7 × 10^−9^ and 5.56 × 10^−9^ M, respectively. The LOD and LOQ values were found lower than the maximum allowable exposure concentration level of NH_3_ in air (35 µg/mL, 2.1 × 10^−3^ M) and NH_4_^+^ in drinking water (0.5 µg/mL, 2.9 × 10 ^−5^ M) set by the US Occupational Safety and Health Administration (OSHA) and the European Association, respectively [42,43]. The probe sensitivity (*S*) towards the NH_4_^+^ ions of the electrode was also computed [38]. The computed *S* value (5.74 × 10^−1^ μA/μM cm^−2^) added further support to the utility of the probe towards NH_4_^+^ detection in real samples.

The analytical features (LOD, LOQ, and linear dynamic range) of the proposed Ads SW–ASV method were successfully compared with the figures of merits of many reported methods [42,43,44,45,46,47,48,49,50,51], as summarized in Table 1 [43,44,45,46,47,48,49,50,51,52]. A comparison of the planned probe revealed a significant improvement in the developed sensing probe and confirmed the good performance and fitness of the probe for NH_3_/NH_4_^+^ detection in water samples.

The developed method was validated via intra- and inter-day analysis of the known ammonium ion concentration (0.05 µg L^−1^) by performing five repeated measurements of NH_4_^+^. In intra-day repeatability, the relative standard deviation (RSD) and the relative error (RE) were found to equal ±1.8 and 3.1%, respectively, close-fitting the precision and accuracy of the probe. Inter-day repeatability of the developed method was also studied by measuring the i_p,a_ of similar fresh solutions over a period of three days. The RSD and RE were found to be less than 2.3 and 3.5%, respectively, supporting the reproducibility and accuracy of the newly developed probe.

### 2.5. Selectivity

The selectivity and reliability of the established probe was tested in the presence of relatively high concentrations of diverse interfering ions in water. An RSD within ±5% of the peak current signal was regarded as the tolerance limit for interfering ions. The developed procedure was applied for the analysis of NH_4_^+^ (2.0 × 10^−6^ M) in the presence of a 1000-fold excess of foreign species, e.g., urea, oxalic acid, acetic acid, and 2-Cl phenol, and the ions Na^+^, K^+^, Zn^+2^, Mn^+2^, Fe^+3^, Al^+3^, PO_4_^−3^, NO_3_^−^, and SO_4_^2−^. Insignificant interference from most common interfering ions was noticed. Thus, the proposed sol–gel/GCE electrode has acceptable selectivity towards the detection of NH_3_/NH_4_^+^ in environmental water samples under the optimized experimental parameters.

### 2.6. Analytical Application

The feasibility of the optimized method was evaluated via analysis of ammonium ions in water samples (tap and Red Sea) after percolation through a 0.45 µm membrane. Water samples were analyzed following the standard addition procedure. In this experiment, standard concentrations (0.01–0.07 µg/mL) of NH_4_^+^ were added to the water samples before treatment and consequently detected following the commended assay. The results are summarized in Table 2, where an excellent recovery (96.0 ± 2.7–101.6 ± 3.1%) of the NH_4_^+^ added was achieved, supporting the analytical utility of the established sensing probe method for the detection of NH_4_^+^ in various water samples. The probe was further validated by measuring the concentration of NH_4_^+^ in spiked tap and sea water samples by the established probe and the official micro-spectrophotometry [15] and the obtained results are summarized in Table 3. A good agreement in terms of accuracy, precision and average recovery was achieved by the established probe and the official method [15]. The calculated Student *t* (*t_exp_* = 0.45–1.25) and *F* (*F* = 1.69–1.78) at 95% confidence (*n* = 5) were lower than the tabulated Student *t* (2.31) and *F* (6.39) [53], respectively. These data added extra evidence to the performance of the proposed probe.

## 3. Conclusions and Future Perspectives

This study reports for the first time the development and characterization of a sol–gel-treated GCE electrochemical sensor based on Berthelot’s reaction. The results validate the integration of a functional sol–gel coating to enhance the sensing performances of the GCE probe towards the facile and rapid detection of NH_3_ and/or NH_4_^+^ in water. The successful detection of ammonia in environmental water samples demonstrates the potential of the developed probe for real world applications. This protocol offered a significant efficiency and selectivity coupled with a short analytical time and excellent reproducibility towards NH_3_/NH_4_^+^ detection. This methodology could also be further improved for the measurement of sub-nM levels of ammonia by combining the on-line enrichment of NH_3_/NH_4_^+^ from large sample volumes of water with supramolecular solvent-based dispersive liquid–liquid microextraction [54] or with the dispersive micro-solid phase micro-extractor (d-µ SPME) packed column [55], and subsequent elution prior to measurements. The development of innovative hierarchically-structured sol–gel coatings with various functionalities and dopants will be of great importance in the electrochemical sensor field.

## 4. Materials and Methods

### 4.1. Chemicals and Reagents

Analytical grade (AG) chemicals and solvents were used as received. Glassware, low density polyethylene (LDPE) bottles, and electrochemical cells were cleaned as reported [22]. A stock solution (1000 mgL^−1^) of NH_4_^+^ was prepared by dissolving 0.2972 g anhydrous NH_4_Cl in ultra-pure water. More diluted working standard solutions (5.6 × 10^−9^–5.6 × 10^−6^ mol. L^−1^) of NH_4_^+^ were prepared by suitable dilutions of the standard stock prior to use. A phenol reagent containing 4% phenol and 0.02% sodium nitroprusside was prepared as reported [22]. Sodium hypochlorite (NaClO) containing 2% (*v*/*v*) of active chlorine was prepared from the commercial stock NaClO (Fluka, 14% active chlorine) by dilution with deionized water, as reported [22]. Phenol and NaClO reagents were kept in well-stoppered standard flasks [22]. A series of Britton–Robinson (B–R) buffer (pH 3–11) were prepared from the acid mixture flasks of boric acid, acetic acid, and phosphoric acid (0.04 M) and by adjusting the solutions’ pH to the required values with NaOH (0.2 M). A series of phosphate buffer (pH 5–10) were also prepared by mixing known concentrations of KH_2_PO_4_ (1.0 × 10^−1^ M) and K_2_HPO_4_ (1.0 × 10^−1^ M) in deionized water to reach the desired pH solution. The sol–gel precursors 3-trimethoxypropylmethacrylate (MAPTMS, 98% assay, Sigma-Aldrich, Dublin, Ireland), zirconium *n*-propoxide (ZPO, 70% in propanol, Sigma-Aldrich, Dublin, Ireland), methacrylic acid (MAAH, 99% assay, Sigma-Aldrich, Dublin, Ireland), 3-aminopropyltriethoxysilane (APTES, 99% assay, Sigma-Aldrich, Ireland), and isopropyl alcohol (IPA, 99% assay, Sigma-Aldrich, Dublin, Ireland) were used as received.

### 4.2. Instrumentation

The particle size of the materials was recorded using a Malvern Nano Zs apparatus. The sol–gel material was prepared 5 times during one week and recorded after 60 min of synthesis. In a quasi-isolated environment, the sols were diluted using isopropanol (IPA, 95:5% *v*/*v*) and filtered through 0.45 µm Whatman syringe filter prior to each run. Samples were run 5 times to permit statistical analysis. FTIR spectra (4000–650 cm^−1^) in the reflection ATR configuration were recorded using a Perkin Elmer GX instrument to assign the vibrational modes of the chemical species within the sol–gel and to assign the influence of the functional silanes on the structure of the reference sol–gel material.

An Autolab PGSTAT302N potentiostat/galvanostat (Metrohm, Barendrecht, The Netherlands) connected to a 663 VA Stand and operated with General Purpose Electrochemical System software (GPES v 4.9007) and Frequency Resonance Analysis software (FRA v 4.9007) was used throughout the study. A conventional three-compartment electrochemical cell was used where the sol–gel-modified GCE served as a working electrode, and a Pt rod and Ag/AgCl were used as auxiliary and standard electrodes, respectively. A JENWAY pH-meter instrument (United Kingdom) equipped with a combined glass–calomel electrode and the Milli-Q Plus system (Millipore, Bedford, MA, USA) was used for pH measurements.

SEM images were recorded employing a Hitachi SU-70 SEM with an accelerating voltage of 2 keV. Prior to analysis, samples were made conductive to minimize charging during image recording. For this purpose, approximately 4 nm of platinium/palladium were coated on the samples using a Cressington 208HR sputter coater. AFM images were recorded using a Pacific Nanotechnology: Nano–RTM to identify the roughness and topography of the sol–gel coatings. Three measurements were taken from three different areas of the same scan size of 80 × 80 µm^2^ of the sample surface. The root-mean-square roughness was measured, and the average roughness value of the surface was calculated.

### 4.3. Fabrication of the Sol–Gel/GCE

The preparation of the sol–gel-modified glassy carbon electrode was carried out as follows:

(i) The sol–gel material was prepared by the combination of MAPTMS and a zirconium complex, prepared from the chelation of ZPO with MAAH, as reported by Cullen et al. [56]. The prepared sol–gel was then functionalized with APTES at different concentrations and diluted in IPA, as illustrated in ESI. 8;
(ii) Prior to modification, bare GCE (2 mm diameter) was refined with alumina (0.05 mm) slurry to a mirror finish and washed with HNO_3_– H_2_O (1/1 *v*/*v*), ethanol, and finally with deionized water and dried with dry air. The GCE was dipped in a small beaker containing 10 mL of treatment sol–gel (1.0% APTES) for 3.0 min. The sol–gel coating was then allowed to dry by evaporation of isopropanol under an IR lamp for 2.0 min.

### 4.4. General Recommended Ads SW–ASV Procedures

The recommended Ads SW–ASV procedures were carried out as follows: To a series of volumetric flasks (10.0 mL), phenol reagent (2.0 mL) and sodium hypochlorite (0.5 mL) solutions were transferred followed by adding known concentrations (5.6 × 10^−9^–5.6 × 10^−6^ M). The solutions were then completed to the mark with the KCl (1.0 × 10^−1^ M) electrolyte. The solutions were kept for 10 min at 25 °C to complete the Berthelot’s reaction. The solutions were stirred, purged with dry N_2_ gas for 15 min, and the stirrer was then stopped. After an equilibrium time of 10 s, the background voltammogram of the supporting electrolyte was then recorded by applying a potential sweep from 0.0 to 1.0 V vs. Ag/AgCl at the optimal deposition potential (0.5 V), accumulation time (50 s), sweep rate (0.07 Vs^−1^), and pulse amplitude (0.05 V). Following these procedures, the impact of the sweep rate (ν = 0.005–0.17 Vs^−1^) on the CVs of NH_4_^+^ (5.55 × 10^−4^ M) using KCl (1.0 M) as the supporting electrolyte was studied.

### 4.5. Analytical Applications

Water (tap and Red Sea) samples were collected and immediately filtered through a cellulose membrane (0.45 µm) and stored in LDPE bottles (500 mL), as previously mentioned [22]. Accurate volumes (2.0 mL) of the pre-filtered water samples were transferred onto a series of volumetric flasks (10.0 mL) containing phenol (2.0 mL) and NaClO (0.5 mL) solutions. The NH_4_^+^ concentration was analyzed by the standard addition method after the addition of known concentrations (5.6 × 10^−7^–3.9 × 10^−6^ M) of NH_4_^+^. The resulting solutions were diluted to 10 mL with KCl as a supporting electrolyte. The flasks were covered for 10 min at room temperature to complete the Berthelot’s reaction, and each solution was then transferred to the cell and analyzed. The i_p,a_ of each solution was then measured at 0.42 V vs. the Ag/AgCl electrode in absence and presence of the standard NH_4_^+^ ions at the optimal conditions. The NH_4_^+^ concentration was then computed using the following equation [38]:

[NH_4_^+^] = [C_stand_] × (i_p,c_)_samp_/(i_p,c_)_stand_ (9)

where [C_stand_] signifies the known NH_4_^+^ concentration, and (i_p,c_)_samp_ and (i_p,c_)_stand_ represent the anodic peak current before and after the standard addition of NH_4_^+^. The recovery percentage of [NH_4_^+^] was then computed using Equation (10):

R, % = [NH_4_^+^]_found_/[NH_4_^+^]_added_ (10)

The unknown NH_4_^+^ concentration could also be computed with the calibration plot using Equation (11):

[NH_4_^+^] = b C_stand_/m. V_x_ (11)

where b and m are the intercept and slope of the standard addition plot, respectively, V_x_ denotes the aliquot volume, and C_stand_ is the NH_4_^+^ concentration.

### 4.6. Validation and Statistical Treatment of the Developed Methodology

Analysis of water samples containing known concentrations (0.01–0.07 µg L^−1^) of NH_4_^+^ was validated using official micro-spectrophotometry [15] as follows: In a volumetric flask (10.0 mL), an accurate volume of NH_4_^+^ was mixed well with trisodium citrate (0.2 M, 0.5 mL), phenol reagent (0.5 mL), and NaClO (0.5 mL). The flask was stoppered, and the contents were stirred for 20 min at ambient temperature over a magnetic stirrer until the formation of deep blue indophenol. Acetic acid (2 M) was added drop by drop to the stirred solution with a micro-syringe until the blue-colored indophenol solution turned red. The volume of the colored aqueous solution was measured up to the mark (10 mL) with deionized water. A 100 μL aliquot of 1-octanol-isooctane (60:40, *v*/*v*) mixed solvent was injected into the aqueous phase, and stirring continued for 2 min to extract the red dye into the organic solvent. The mixture was kept for about 10 min when the colored extract layered above the aqueous phase. The organic phase containing the red indophenol was placed in an insert vial and mixed with 5 drops of 0.8 M NaOH until the indophenol partitioned into the aqueous alkali as its blue form. The insert vial was placed in a centrifuge tube and centrifuged for 2 min for phase separation. The blue dye at the bottom of the aqueous phase was withdrawn using a syringe, and the absorbance was measured at 630 nm against the blank.

The averages ± standard deviations of the replicates (*n* = 3) were analyzed by SPSS V.13 (SPSS Inc., Chicago, IL, USA). One-way ANOVA and the least significant difference (LSD) at *p* < 0.05 were performed. The obtained results of the within-bottle mean square (MS_within_) and between-bottle mean square (MS_among_) were computed using Equations (12) and (13) [52].

(12) $$S_{\mathrm{bb}}=\sqrt{\frac{MS_{\mathrm{among}}-MS_{\mathrm{within}}}{n}}$$

(13) $$u_{bb}=\sqrt{\frac{MS_{\mathrm{within}}}{n}}\cdot \sqrt[{4}]{\frac{2}{\upsilon_{MS_{\mathrm{within}}}}}$$

where $\upsilon_{MS_{\mathrm{within}}}$ is the freedom of MS_within_, and S_bb_ and U_bb_ are the variances between-bottle and within-bottle, respectively.

## Figures and Tables

**Table 1:** A comparison between the figures of merits of the developed sensing probe (sol–gel/GCE) and other spectrochemical and electrochemical assays.

Detection Technique	LDR	LOD	Sample Type	Ref.
Potentiometry	20 µM–100 mM	12 µM	Real sample	[44]
Conductometry	(0–8) mM	10 nM	Artificial water sample	[45]
Square wave voltammetry	(4.2–66) µM	0.12 mM	Experimental environment	[46]
Impedance spectroscopy	(0.1–1000) mM	1 µM	River	[47]
Square wave voltammetry	(0.0–60) µM	3.17 µM	Aqueous solution	[43]
Square wave voltammetry	(0.56–5.3) mM	0.233 mM	-	[48]
Square wave voltammetry	(10–1000) µM	6.23 µM	Drinking water	[49]
Manual operation with a hand-held fluorescence detector	Up to 5 µM	3.5 nM	Fresh water and sea water	[18]
Spectrophotometry	(0.1–0.7) mM	0.7 µM	Lake, river, and ground water	[50]
Fluorometry	8 nM–0.556 µM	3 nM	Natural water sample	[51]
Ads SW–ASV	5.0–100 µM	0.03 µM	Environmental water samples	[52]
Ads SW–ASV	5.56 nM–5.56 µM	1.7 nM	Tap and sea water	This work

**Table 2:** Analytical data for trace determination of NH_4_^+^ in water samples by the developed probe.

Sample	Spiked (µg/mL)	Found (µg/mL) ^a^	Recovery (%) ^b^
Tap water	0.0	ND	--
	0.05	0.048 ± 0.0013	96.0 ± 2.7
	0.07	0.068 ± 0.002	97.14 ± 2.9
Sea water	0.0	0.0062 ± 0.0012	--
	0.05	0.057 ± 0.0016	101.6 ± 3.1
	0.07	0.074 ± 0.0024	96.9 ± 4.7

ND = not detected. ^a^ Average of measurements (*n* = 5) ± standard deviation. ^b^ Mean recovery (*n* = 5) ± RSD.

**Table 3:** Analysis of NH_4_^+^ in water samples by the proposed probe (**A**) and micro-spectrophotometry (**B**) [15].

Sample	Added (µg/mL)	Found (µg/mL) ^a^		Recovery (%) ^b^		Student *t* Test (2.31) *	*F* (6.39) *
		A	B	A	B		
Tap water	0.03	0.0315 ± 0.002	0.031 ± 0.0015	105.0 ± 6.3	103.3 ± 4.8	0.45	1.78
Sea water	0.03	0.037 ± 0.0017	0.032 ± 0.0013	102.7 ± 5.5	106.7 ± 4.1	1.25	1.69

^a^ Mean ± standard deviation (*n* = 5). ^b^ Mean recovery ± RSD (*n* = 5). * Figures in the parenthesis are the tabulated Student *t* and *F* values at 95% (*p* = 0.05) confidence.

## Data Availability

The data presented in this study are openly available in the article. All data and materials are offered on demand from the principal authors.

## Supplementary Materials

The following supporting information can be downloaded at: https://www.mdpi.com/article/10.3390/gels10060382/s1, ESI. 1: A mechanism describing the formation of the blue colored product of the Berthelot’s reaction (Indophenol); ESI. 2: FTIR spectrum of APTES sol gel; ESI. 3: Plots of ip,a versus square root of sweep rate (v) of bare GCE (a) and Sol-Gel/GCE (b) working electrodes of K3[Fe (CN)6] solution-KCl electrolyte; ESI. 4: The plot of log ip,a vs. log ν of of indophenol in the presence of ammonium ions (5.55 × 10^−4^ M) in KCl (1M); ESI. 5: The plot of ip, a versus sweep rate (v) of indophenol in the presence of NH4+ ions (5.55 × 10^−4^ M) in KCl (1.0 M) at pH 10; ESI. 6: Ads SW-ASW of indophenol in the presence of ammonium ions (2.8 × 10^−6^ M) in different supporting electrolytes at Sol gel/GCE vs. Ag/AgCl reference electrode at 50 mVs^−1^ sweep rate; ESI. 7: Effect of dipping time on the oxidation peak current of indophenol in presence of NH4+1 (2.8 × 10^−6^ M) at 1M KCl; ESI. 8: A scheme describing the four steps of the preparation of the reference hybrid Sol-gel material by Oubaha, 2019 [36].
