# Supplementary material for: Development and Characterization of a Sol–Gel-Functionalized Glass Carbon Electrode Probe for Sensing Ultra-Trace Amounts of NH3 and NH4+ in Water
Source: Gels. 2024 Jun 4;10(6):382. doi: 10.3390/gels10060382 (PMC11203079; doi:10.3390/gels10060382)
Supplement: Supplementary file 1 [file gels-10-00382-s001.zip › gels-3027960-supplementary.pdf]

Electronic Supplementary Information's (ESI)

Manuscript Title:

Development and characterization of a sol-gel functionalized glass carbon electrode probe for sensing ultra-trace amounts of NH_3 and NH_4^+ in water

H. Alwael [1‡], M. Oubaha [2] and **M. S. El-Shahawi** [1‡ *]

[1]Department of Chemistry, Faculty of Science, King Abdulaziz University, P.O. Box 80203, Jeddah 21589, Saudi Arabia

[2]. Centre for Research in Engineering Surface Technologies (CREST), Technological University Dublin, FOCAS Institute, 13 Camden Row, Dublin 8, Ireland

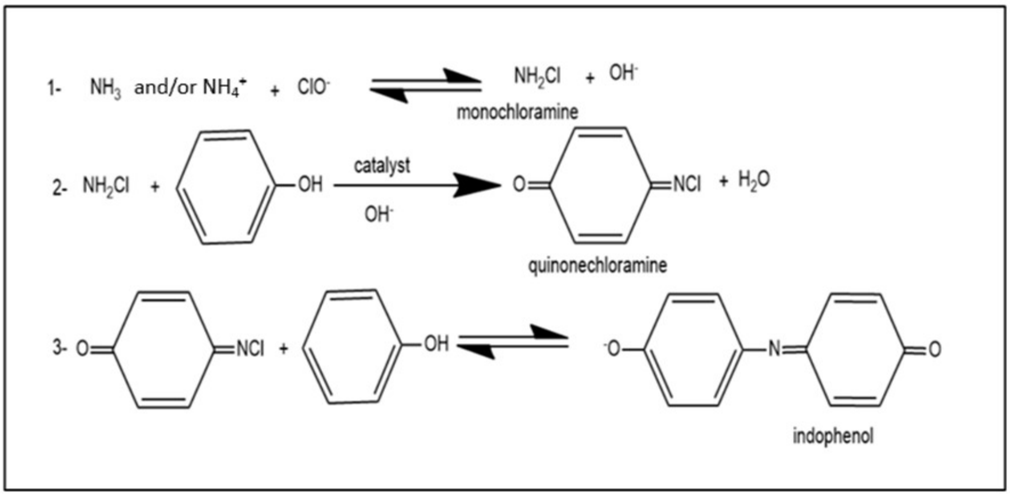

ESI. 1. A mechanism describing the formation of the blue colored product of the Berthelot's reaction (Indophenol).

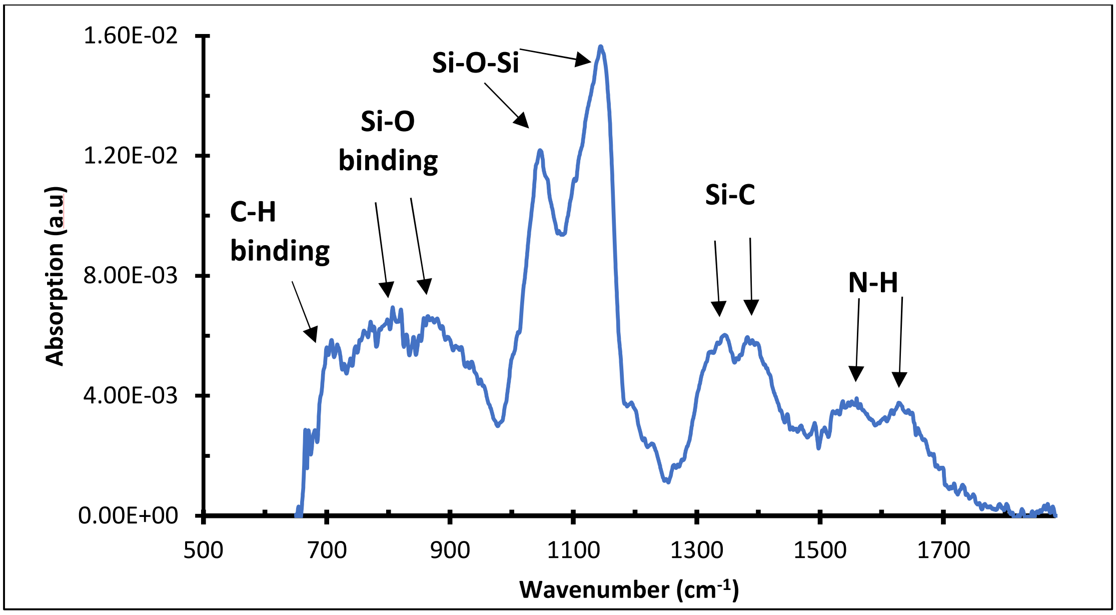

ESI. 2. FTIR spectrum of APTES sol gel.

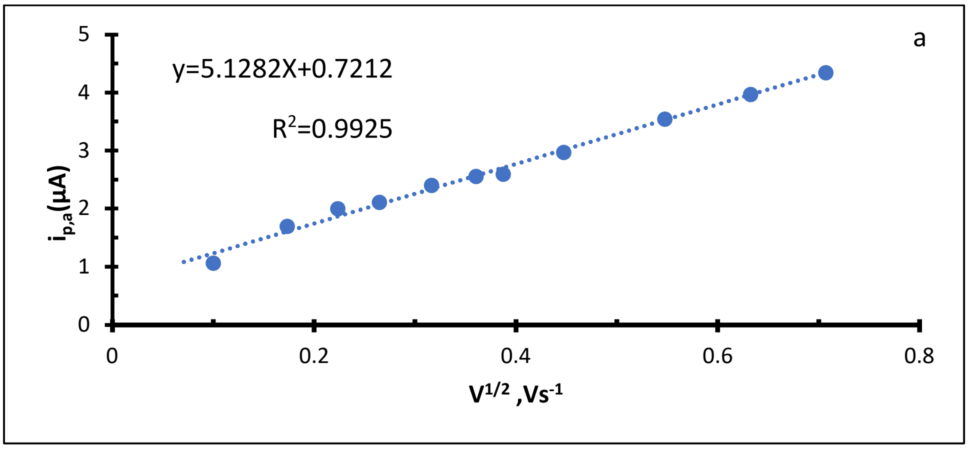

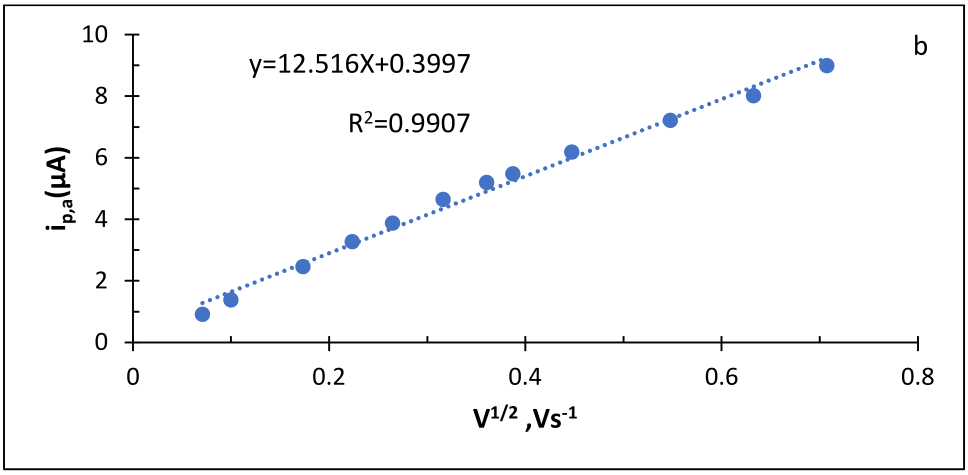

ESI. 3. Plots of ip,a *versus* square root of sweep rate (v) of bare GCE (a) and Sol-Gel/GCE (b) working electrodes of $K_3[Fe(CN)_6]$ solution-KCl electrolyte.

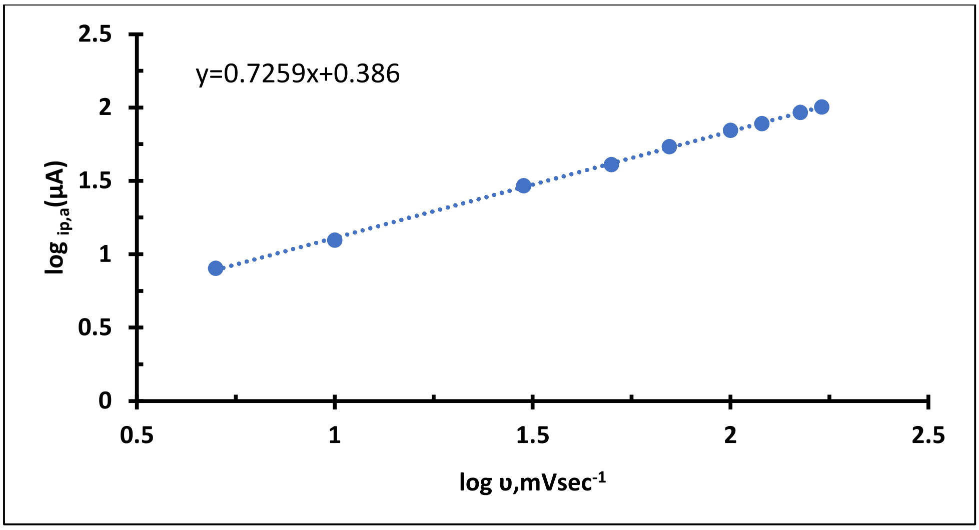

ESI. 4. The plot of log $i_{p,a}$ *vs.* log ν of indophenol in the presence of ammonium ions (5.55×10^{-4} M) in KCl (1M).

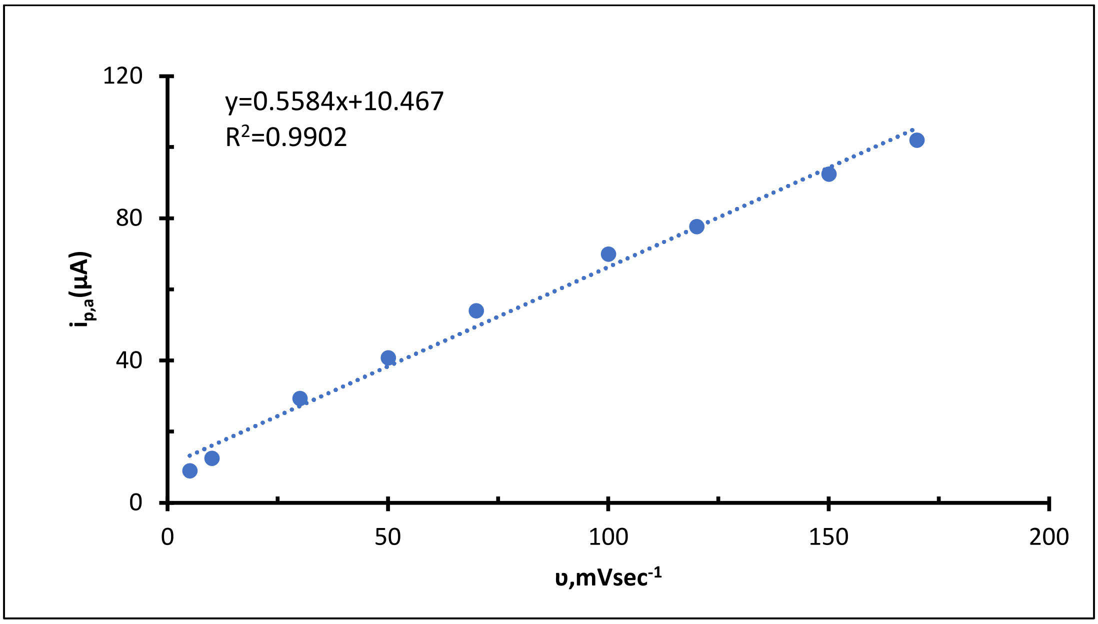

Fig. ESI. 5. The plot of $i_{p,\,a}$ *versus* sweep rate (ν) of indophenol in the presence of NH_4^+ ions (5.55×10^{-4} M) in KCl (1.0 M) at pH 10.

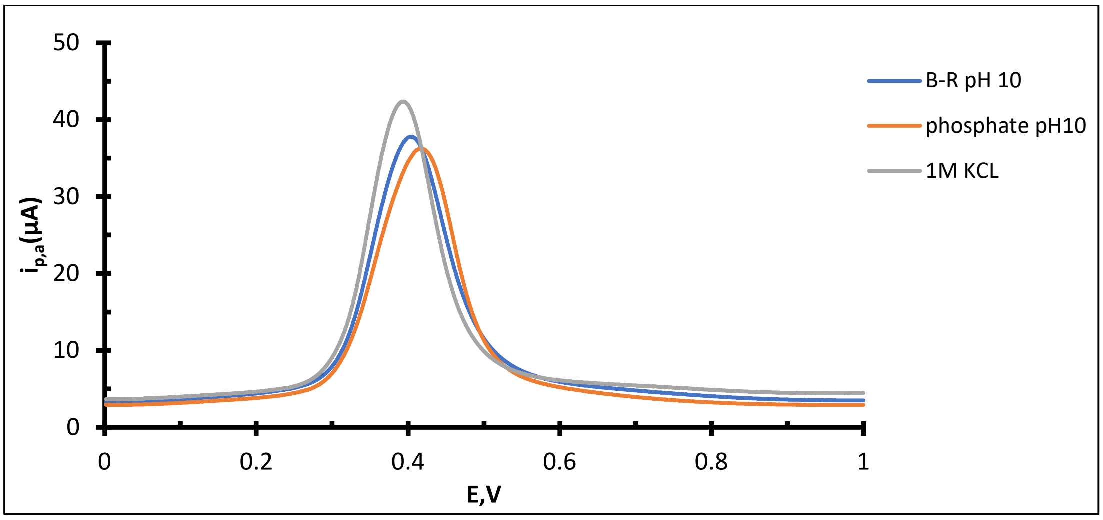

ESI. 6. Ads SW-ASW of indophenol in the presence of ammonium ions (2.8×10^{-6}M) in different supporting electrolytes at Sol gel/ GCE vs. Ag/AgCl reference electrode at 50 mVs^{-1} sweep rate.

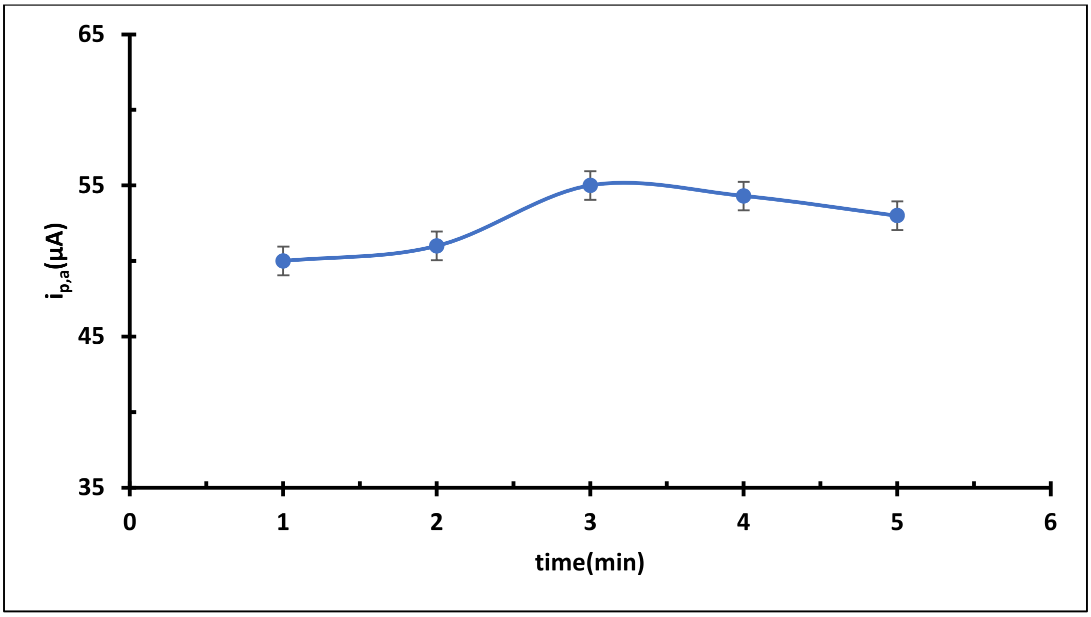

Fig. ESI. 7. Effect of dipping time on the oxidation peak current of indophenol in presence of NH_4^{+1} (2.8×10^{-6} M) at 1M KCl.

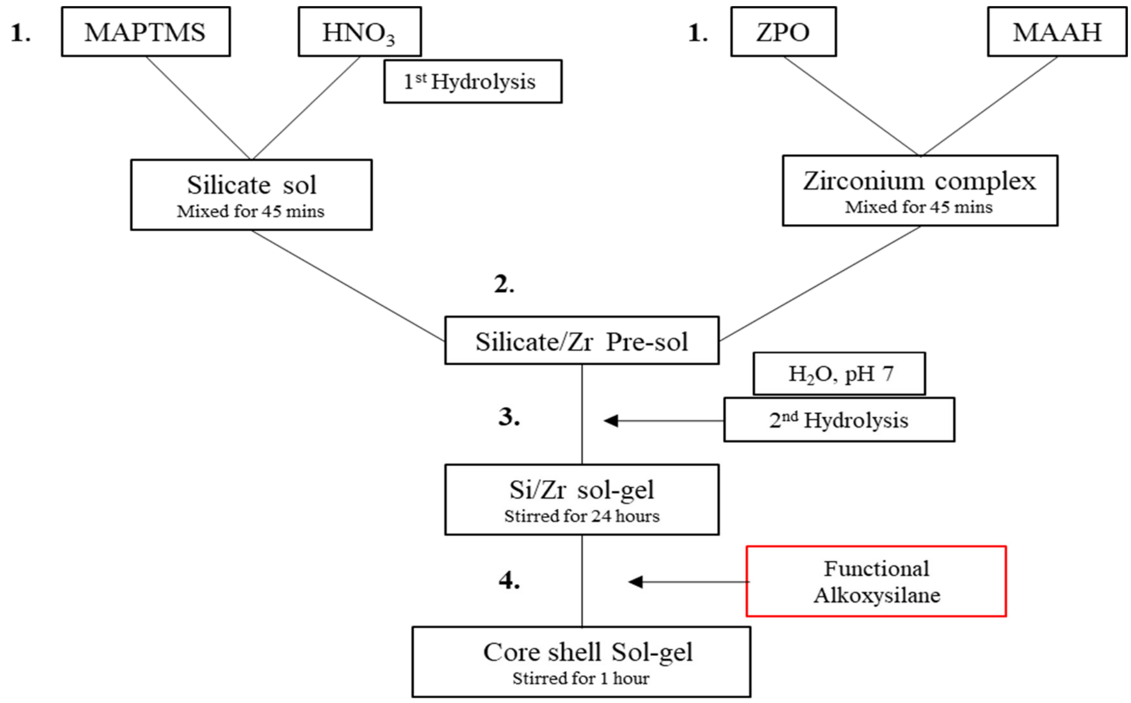

ESI. 8. A scheme describing the four steps of the preparation of the reference hybrid Sol-gel material by Oubaha, 2019 [36].
